# Changes in the volatile components of squid (*illex argentinus*) for different cooking methods via headspace–gas chromatography–ion mobility spectrometry

**DOI:** 10.1002/fsn3.1877

**Published:** 2020-09-12

**Authors:** Zhenkun Cui, Han Yan, Tatiana Manoli, Haizhen Mo, Hongbo Li, Hao Zhang

**Affiliations:** ^1^ School of Food Science Henan Institute of Science and Technology Xinxiang China; ^2^ Faculty of Food Technologies Sumy National Agrarian University Sumy Ukraine; ^3^ Faculty of Technology and Commodity Science of Food Products and Food Business Odessa National Academy of Food Technologies Odessa Ukraine; ^4^ School of Food and Bioengineering Shaanxi University of Science and Technology Weiyang University Campus Xi'an China

**Keywords:** aroma, cooking method, GC‐IMS, sous vide, Squid, volatile components

## Abstract

Squid products are becoming more and more popular with consumers because of their high yields and nutrition, including novel textures with desirable sensory properties. However, it has not been determined whether the cooking method has effects on the flavor of the squid. In this study, the aroma and volatile substances of squid samples from different cooking methods (boiled, steamed, sous vide) were determined and analyzed by headspace–gas chromatography–ion mobility spectrometry and differentiated by using, as well, an electronic nose and sensory evaluation. A total of 43 characteristic flavor compounds were identified. Based on the signal intensity of the identified violate compounds, we established a fingerprint of heat‐treated squid from different cooking methods. Due to the long‐term low‐temperature heating conditions under vacuum, the flavor of sous vide squid is different from steamed and boiled squid, and it has unique special flavor compounds. Different cooking methods can affect the aroma of squid, providing support for the industrial production of squid.

## Graphical abstract

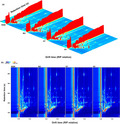

## 1 INTRODUCTION

*Illex Argentines*, the Argentinian squid, is the most important cephalopod species and major component of commercial squid in the southwest Atlantic (Waluda, Rodhouse, Podestá, Trathan, & Pierce, 2001). The volume of cephalopods, including squid, cuttlefishes, and octopuses, harvested and consumed in the world during 2017 was 3,772,567 t (data from FISHSTAT, FAO). The edible portion of squid is as high as 80%, which is about 20% higher than that of fish in general. As a kind of invertebrate, the muscle protein of squid is slightly different from that of ordinary fish. The muscle protein of squid contains some paramyosin that has no ATPase activity to influence the protein stability, gel strength, and other properties of squid products (Mirzaei & Regnier, 2006), limiting the processing of squid. At present, research on the processing technology of squid can be divided into two categories. One is the optimization and improvement of traditional squid processing technology, such as squid rings (Tomac, Cova, Narvaiz, & Yeannes, 2017), dried squid (Dong, Zhu, Li, & Li, 2013), and frozen squid products (Gou, Lee, & Ahn, 2010). The other is the comprehensive processing and utilization of squid by‐products or minced meat leftovers to extract biologically active peptides (Alemán, Giménez, Pérez‐Santin, Gómez‐Guillén, & Montero, 2011; Alemán, Gómez‐Guillén, & Montero, 2013) or squid flavor condiments (Lyberg & Adlercreutz, 2008).

Sous vide cooking (SV) is a particular cooking method in which the raw materials are placed in a vacuum bag and placed in water with precise temperature (55–90°C) control heating for a long time (Schellekens, 1996). SV differs from traditional cooking methods, where vacuum packaging prevents food oxidation, reduces flavor and moisture loss during cooking, prevents the growth of aerobic bacteria, and improves shelf life (Baldwin, 2012). Additionally, precise temperature controlling can maintain the color of foods. Christensen et al. (Christensen et al., 2013) found that low‐temperature and long‐time (LTLT) heating can reduce the strength of connective tissue and increase the solubility of collagen, which makes beef as tender as veal. Roldán, Antequera, Martin, Mayoral, and Ruiz (2013) found the lamb loin tenderness positively correlated with cooking time (6, 12 hr, and 24 hr). However, as the cooking temperature increased, the weight loss of lamb loin increased, and the moisture content decreased. SV can improve lamb loin brightness and redness, as well as ensure biosafety at 60°C. Moreover, beef cooked by SV (59°C, 5 hr) did not exhibit an increase in thiobarbituric acid reactive substances (TBARS) and warmed‐over flavor (WOF) after 30 d of storage (Hansen, Knøchel, Juncher, & Bertelsen, 1995). Many scholars have also evaluated the safety of SV. After cooking at 50–62°C, the number of mesophiles and psychrotrophic bacteria in beef decreased significantly (Botinestean, Keenan, Kerry, & Hamill, 2016; Hansen et al., 1995; Vaudagna et al., 2002). Moreover, the number of Escherichia coli and mesophiles in pork cooked at 53°C for several hours also decreased significantly (Becker, Boulaaba, Pingen, Röhner, & Klein, 2015; Salaseviciene, Vaiciulyte‐Funk, & Koscelkovskienė, 2014). To reduce the nutrient loss while optimizing overall quality and shelf life of meat during cooking, lots of food processors use SV technique to replace traditional cooking methods, such as frying, microwaving, and grilling (del Pulgar, Gazquez, & Ruiz‐Carrascal, 2012; Roldan, Antequera, Perez‐Palacios, & Ruiz, 2014). Thus far, scholars have only focused on SV cooking technology for the protection of food nutrients, quality improvement, and food safety. In addition, they think that the lower heating temperature of SV produces less flavor (Calkins & Hodgen, 2007; Cross, Stanfield, & Koch, 1976). Therefore, there are fewer studies on the flavor of SV. The flavor characteristics of squid by different cooking methods have yet to be reported.

Ion mobility spectrometry (IMS) is a technology that analyzes trace chemical substances based on differences in the migration speed of different ions in the gas field in an electric field. IMS is a chemical substance analysis technology developed in the late 1960s and early 1970s (Eiceman, Karpas, & Hill, 2013). The most prominent advantages of IMS are that it can ionize analytes at atmospheric pressure, and the detection limit is as low as ng/L (Armenta, Alcala, & Blanco, 2011). IMS has been applied to the detection of drugs (Verkouteren & Staymates, 2011), chemical warfare agents (Rearden & Harrington, 2005), and explosives (Asbury, Klasmeier, & Hill, 2000). With the development and improvement of IMS technology, it has gradually been applied in food inspection, such as food adulteration (Garrido‐Delgado, Muñoz‐Pérez, & Arce, 2018), determination of food quality (Ivanov, Bilgucu, Ivanova, & Dimitrova, 2020; Snyder, Harden, Davis, Shoff, & Maswadeh, 1995), food process control (Karpas, Guamán, Calvo, Pardo, & Marco, 2012), and chemical food safety (Gloess, Yeretzian, Knochenmuss, & Groessl, 2018).

Therefore, the main objective of the present study was to analyze by GS‐IMS the volatile components of squid and differentiate the samples based on their volatile compound profile, using, as well, an electronic nose and sensory evaluation. Through principal component analysis, the establishment of fingerprints, and heat map analysis, the differences and correlations of volatile flavor substances in squid from different cooking methods were explored. The results provide a foundation for the replacement of traditional cooking methods by SV technology.

## 2 MATERIALS AND METHODS

### 2.1 Sample preparation

The Argentinian squid (approximate net weight 400 g) was purchased from an aquatic products market in Qingdao city, Shandong Province of China. The squid specimens were kept refrigerated with flake ice inside polystyrene boxes provided with a lid and holes for drainage. Samples were transported to the laboratory at −18°C. Prior to cooking, squid specimens were separated into the head, foot (wrist), and ketone body with scissors after thawing and washing. The average weight of the ketone body of squid was 20 ± 4 g (*n* = 16). Each squid specimen length, width, and thickness of the ketone body were 4 ± 1 cm, 4 ± 1 cm, and 1 ± 0.4 mm, respectively. All squid were randomly divided into four groups to prepare for processing and kept at 4°C until further analysis. From each specimen, one specimen remained raw (RAW) as a control, and the others were subjected to cooking methods.

### 2.2 Cooking methods

Different cooking methods were selected as the preparation procedures for squid samples in this study: boiling (BO), steaming (ST), and sous vide (SV).

#### 2.2.1 BO method

The squid samples were placed in a stainless steel pot of boiling water (2 L) for 5 min. After cooking, the samples were removed and drained on absorbent papers.

#### 2.2.2 ST method

The squid samples were placed in a steam oven (SCC WE 61, Germany Rational) at 100°C for 5 min. After cooking, the samples were removed and drained on absorbent papers.

#### 2.2.3 SV method

The samples were put into a plastic vacuum bag (nylon/polyethylene pouches, operating temperature of −20°C/121°C) and sealed using a vacuum sealer (DZ‐260, Dajiang Holding Group Electric Co. LTD, China). The samples then were cooked in temperature‐controlled water bath (60°C) for 30 min (Cui, Dubova, & Mo, 2019).

All the samples were soaked in ice water after cooking for 30 min and stored at −20°C until analysis, including the control group.

### 2.3 Sensory evaluation

The sensory evaluation team consisted of 20 people, between 18 and 25 years old, all of whom had undergone sensory evaluation training. A hedonic scale method from 1 to 9 points was adopted, where 1 means extremely disliked and 9 means extremely liked.

### 2.4 Electronic nose analysis

The electronic nose (PEN 3, Germany AirSense) was used to preliminarily evaluate the aroma profile of the squid samples. It was conducted by the following procedure. Taking the center of the squid sample to be tested, each sample (1 g) was prepared in a 20 ml gas chromatographic analysis bottle at room temperature for 30 min. The injection rate was 600 ml/min; the carrier gas flow rate was 600 ml/min; and the measurement time was 60 s. Thereafter, the sensors were purged by clean dry air for 180 s. The parameters were optimized, and each analysis was repeated 3 times.

The types of sensitive substances corresponding to the ten sensors of the electronic nose are as follows: W1C: aromatic hydrocarbon compounds; W5S: nitrogen oxide compounds; W3C: ammonia, aromatic molecules; W6S: hydride; W5C: olefins, aromatic, polar molecules; W1S: alkanes; W1W: sulfur compounds; W2S: alcohols, partially aromatic compounds; W2W: aromatic compounds, sulfur organic compounds; and W3S: alkanes and fats.

### 2.5 Determination of volatile components by HS‐GC‐IMS

#### 2.5.1 Isolation of volatile organic compounds in squid

Volatile compounds of squid from different cooking methods were identified by a GC‐IMS flavor analyzer (FlavourSpec®, Dortmund, Germany). Each squid sample (2 g) was placed in a 20 ml headspace vial and sealed. Samples were subsequently incubated at 60°C for 15 min. Finally, 500 μL headspace was injected automatically via a heated syringe (65°C) into the heated injector of the GC‐IMS equipment under conditions reported below. Three parallel samples were analyzed from the same processing method.

#### 2.5.2 Chromatographic conditions

The gas chromatographic separation was performed at 60°C on a FS‐SE‐54‐CB‐1 capillary chromatographic column (15 m × 0.53 mm, 1 μm). High‐purity nitrogen (99.99%) was as a carrier gas with a flow rate of 150 ml/min and a programmed flow as follows: 2 ml/min for 2 min; linear increase to 100 ml/min over 18 min; and total run time of 20 min. To avoid cross‐contamination, the syringe automatically flushed for 30 s with nitrogen gas before each analysis and 5 min after each analysis.

The n‐ketones C4–C9 (Sinopharm Chemical Reagent Beijing Co., Ltd, China) were employed as external references to calculate the retention index (RI) of each volatile compound. By comparing the RI and drift time (DT) with the GC × IMS Library Search (FlavourSpec®, Dortmund, Germany), the volatile compounds in squid samples from different cooking methods were identified. The signal intensity represents the height or the peak area. Using laboratory analytical viewer, reporter, gallery plot, and GC × IMS Library Search database supported by HS‐GC‐IMS instrument, three‐dimensional (3D) and two‐dimensional (2D) fingerprint maps of the volatile organic components of squid samples were constructed.

### 2.6 Data analysis

The sensory data analysis was conducted using SPSS 13.0 software (IBM). Principal component analysis (PCA) of electronic nose was carried out with Win Muster software. Volatile organic components (VOCs) were analyzed by laboratory analytical viewer (LAV) and GC × IMS Library Search (FlavourSpec^®^).

## 3 RESULTS

### 3.1 Sensory evaluation

**Figure 1:**
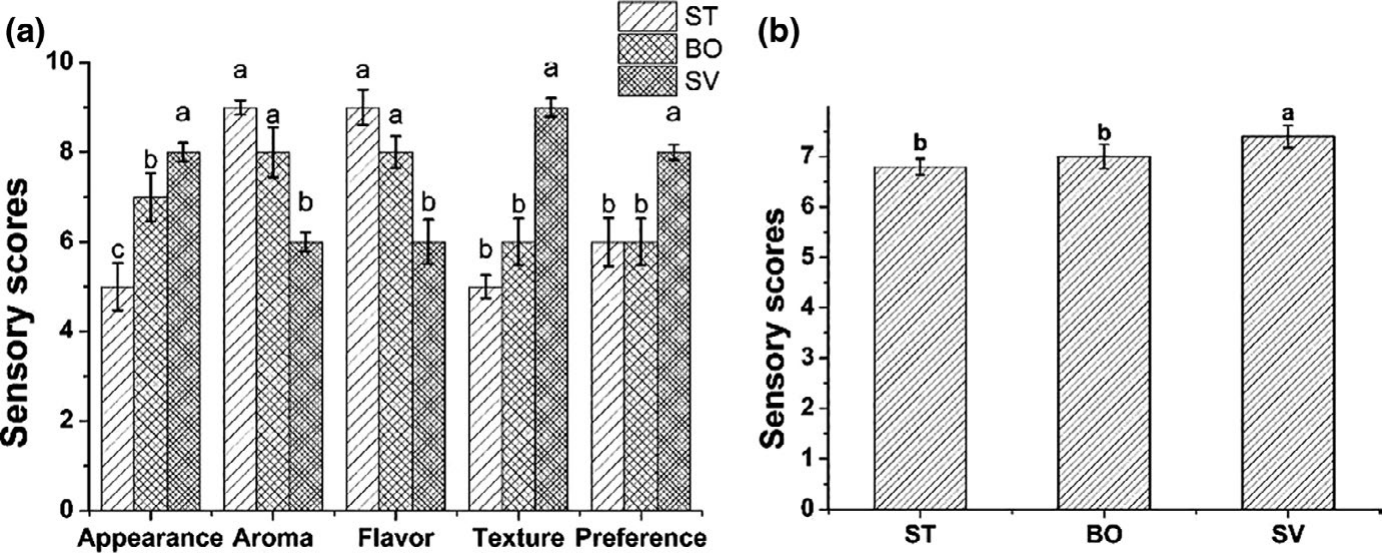
Sensory scores of squid in different cooking methods. a, Sensory scores of appearance, aroma, flavor, texture, and preference of squid prepared from different cooking methods. b, Comprehensive sensory score of squid from different cooking methods. BO: boiled squid; ST: steamed squid; SV: sous vide cooking squid

Sensory analysis of squid samples from different cooking methods was performed. Different cooking methods have different sensory evaluations of squid (Figure 1). The squid cooked by ST and BO contracted and curled to varying degrees, while the squid cooked by SV contracted less and did not curl. There was no difference in the color of the squid processed by the three cooking methods. In terms of texture, the SV sample was excellent and the softest. In terms of aroma and flavor, ST and BO were very outstanding, with a strong aroma of cooked squid. The SV samples had the best score in appearance, texture, and preference, while the ST samples performed best in terms of aroma and flavor. The comprehensive sensory score of SV samples was the best because of their tender texture and smoother appearance. However, the aroma and flavor of the SV samples were obviously insufficient and different from ST and BO. Further research is needed.

### 3.2 Analysis of flavor substances of squid via electronic nose

**Figure 2:**
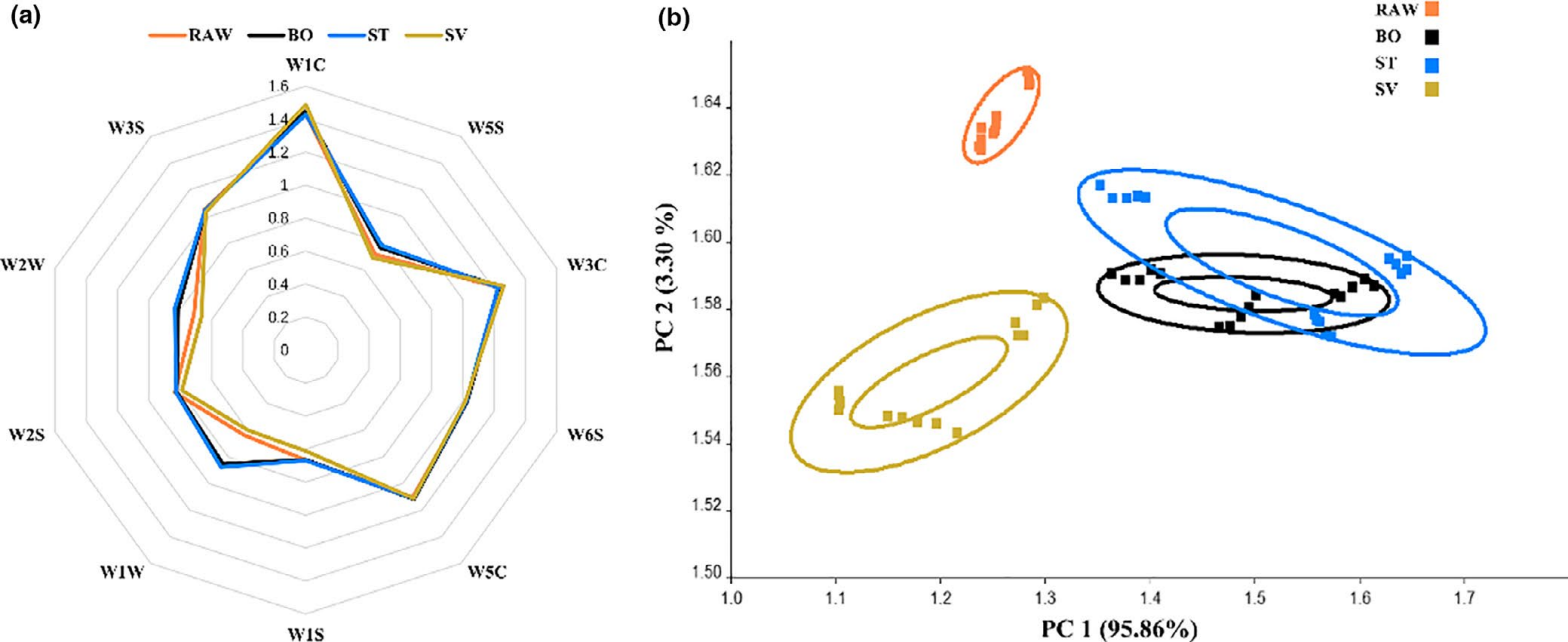
Electronic nose analysis. a, Radar charts of volatile compounds in squid from different cooking methods. b, Analysis of the predominant volatile compounds in squid samples. RAW: raw squid; BO: boiled squid; ST: steamed squid; SV: sous vide cooking squid

Compared with traditional sensory analysis, electronic nose is simple, fast, objective, and intuitive. According to the different response values of the electronic nose sensor to the aroma components of squid in different cooking methods, an intuitive radar chart was established, as shown in Figure 2a. The radar chart analysis method is mainly employed to study the sensors. Using this method, the contribution rate of each sensor to the squid sample can be distinguished, so as to investigate which type of volatile components play predominant roles in distinguishing the sample. From Figure 2a, the difference in volatile flavor compounds from different squid samples was mainly in the sensors W2W, W5S, W1W, and W1C, which are aromatic compounds, sulfur organic compounds, and nitrogen oxide compounds. It can be seen from the radar chart that the flavor of squid is greatly influenced by aromatic compounds. Conversely, the BO and ST squid samples had more obvious responses at W1W and W2W, which can be contributed to sulfide. Specific volatile components need further verification.

In order to highlight the aroma differences in squid from different cooking methods, a PCA scatter diagram (Figure 2b) was generated according to electronic nose data of the overall flavor substance composition. PCA is a multivariate statistical analysis technique that examines the correlation between multiple variables and reveals the internal structure between multiple variables through a few principal components (Jo et al., 2013). Generally, the PCA model is selected as the separation model when the cumulative contribution rate reaches 60% (Wu et al., 2015). PCA of aromatic substances from different heat‐treated squids (5 detection signals from 48s to 52s) was performed, and the result shows that the first principal component contribution rate was 95.86%; the second principal component contribution rate was 3.30%; and the cumulative contribution rate was 99.16%. This indicates that the two principal components can adequately represent predominant characteristics of the sample. Each sample in the group was relatively concentrated in a specific range and has a precise distance from the clustered areas of other groups. RAW samples and SV samples were clustered separately (the farthest distance in the PCA chart), while BO and ST were clustered together. This suggests that these methods have a high degree of similarity. Moreover, the results indicate that different cooking methods lead to differences in squid aroma.

### 3.3 Differences in VOCs in squid samples

**Figure 3:**
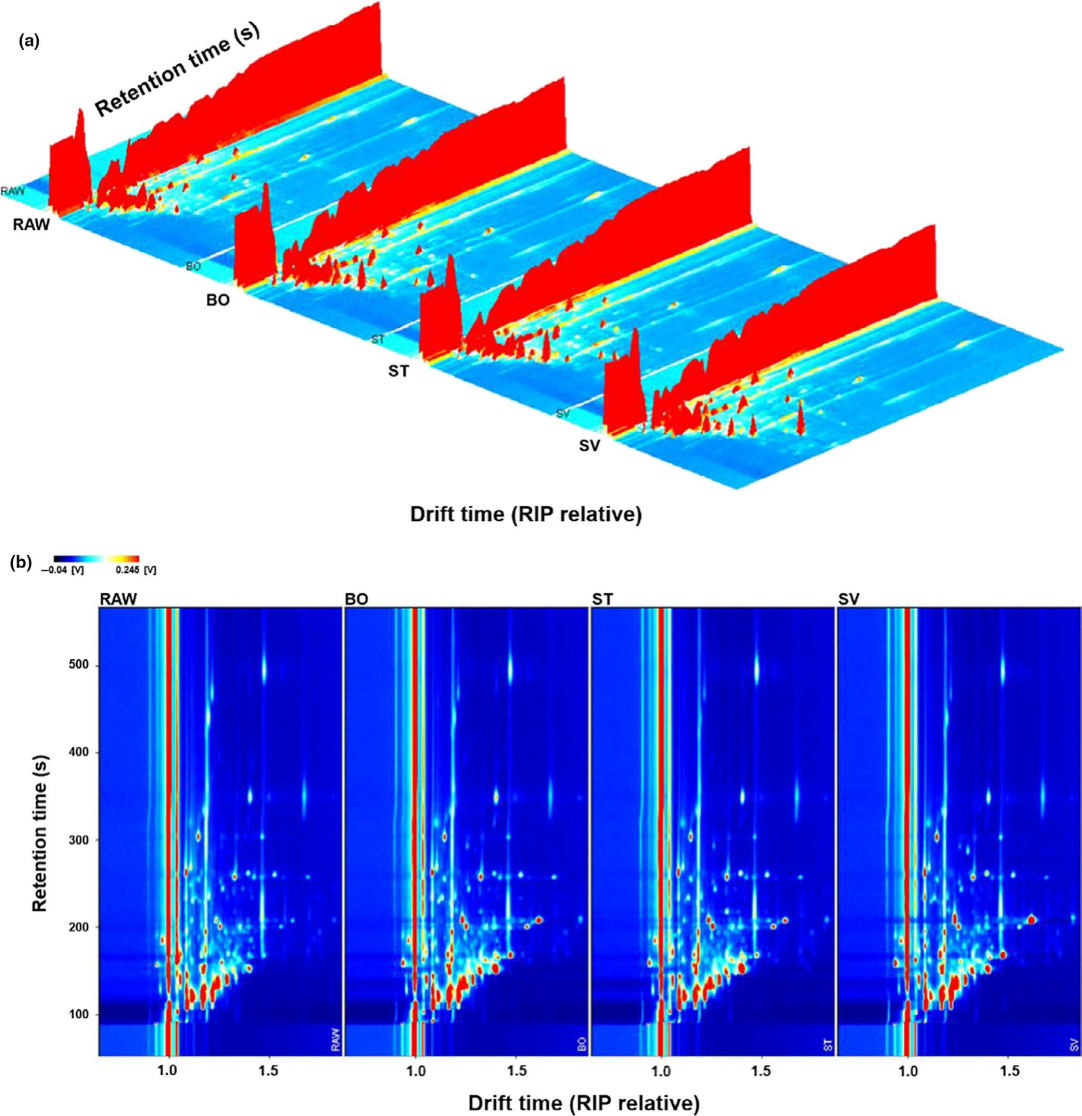
Gas chromatography–ion mobility spectrometry of squid from different cooking methods. a, Three‐dimensional topographic plots and chromatograms of squid samples. The y‐axis represents the retention time of the gas chromatograph; the x‐axis represents the ion migration time for identification, and the z‐axis represents the peak height for quantification. b, Two‐dimensional topographic plots and chromatograms of squid samples. RAW: raw squid; BO: boiled squid; ST: steamed squid; SV: sous vide cooking squid. The ordinate represents the retention time, and the abscissa represents the migration time. The red vertical line represents the reaction ion peak (RIP), and the migration time after normalization was 8.0 ms. Each point on the right of RIP represents a volatile substance. Color represents the signal intensity of the substance. White indicates low intensity, and red indicates high intensity. The darker the color, the greater the intensity

A series of physical and chemical changes occur after meat is heated, which significantly affects the quality of the processing (Shahidi, Rubin, D'Souza, Teranishi, & Buttery, 1986). HS‐GC‐IMS analyzed the differences between volatile compounds in heat‐treated squid samples. Figure 3a shows the 3D topographic plot spectrum of volatile flavor compounds. From Figure 3a, the VOCs of squid from different cooking methods are very similar, but the signal intensities are slightly different. After heat treatment, the content of most flavor compounds changes to varying degrees.

Although the differences in volatile flavor compounds can be visualized in the 3D spectrum, a 2D plot spectrum is more evident. Figure 3a shows a top view of the 3D GC‐IMS spectrum of Figure 3b projected onto a 2D plot, which can directly compare the differences in flavor substance for various heat‐treated squid. However, a volatile compound may produce one or more bright spots, representing monomers or dimers and trimers, depending on the concentration of volatile compounds. The entire spectrum represents all components found in the headspace sample. Most of the signals in the topographic plot of squid samples from different cooking methods appeared between 100 and 200 s, while in SV samples the signals were distributed between 100 s and 300 s. Moreover, the signal intensity was stronger than that the other heat treatments.

### 3.4 Identification of VOCs in squid from different cooking methods

Figure 3 shows the differences in VOCs in squid prepared from different cooking methods intuitively, but it is difficult to accurately determine the specific substance in the 2D and 3D spectrum maps. Through GC‐IMS separation, the differences in volatile substances in the squid samples are elucidated. According to the retention time and ion migration time of volatile substances in gas chromatography, 99 volatile substances were detected, and 43 specific volatile substances were determined (Table 1). Due to different concentrations of VOCs, certain compounds might produce multiple signals or spots (dimers or trimers).

**Table 1:** Identified compounds in squid prepared from different cooking methods

**No.**	**Compound**	**CAS**	**Molecule Formula**	**MW**	**RI** ^a^	**Rt** ^b^	**Dt** ^c^	**Comment**
1	1C	unidentified	*	0	370.2	89.741	0.9376	
2	Ethanol	C64175	C2H6O	46.1	433.3	103.409	1.0443	
3	2C	unidentified	*	0	384.3	92.786	1.0901	
4	Acetone	C67641	C3H6O	58.1	519.7	122.144	1.1168	
5	2‐butanone	C78933	C4H8O	72.1	604.1	140.442	1.0581	mono
6	Isopropyl alcohol	C67630	C3H8O	60.1	514	120.912	1.1701	mono
7	3C	unidentified	*	0	548.4	128.353	0.9589	
8	4C	unidentified	*	0	682.4	159.787	0.9414	
9	5C	unidentified	*	0	629.6	146.145	1.0412	
10	Acetic acid ethyl ester	unidentified	*	0	622.6	144.529	1.092	mono
11	6C	unidentified	*	0	753.9	185.645	0.968	
12	1‐propene−3‐methylthio	C10152768	C4H8S	88.2	694.8	163.592	1.0421	
13	7C	unidentified	*	0	739.1	179.623	1.0888	
14	3‐hydroxybutan−2‐one	C513860	C4H8O2	88.1	720.4	172.421	1.0573	mono
15	8C	unidentified	*	0	778.2	196.065	1.0896	
16	9C	unidentified	*	0	766.6	191.051	0.94	
17	10C	unidentified	*	0	775.8	195.031	1.1854	
18	11C	unidentified	*	0	806.3	208.671	1.1153	
19	Prop−1‐ene−3,3'‐thiobis	C592881	C6H10S	114.2	853.3	231.52	1.119	
20	12C	unidentified	*	0	859.2	234.571	1.0879	
21	3‐methylthiopropanal	C3268493	C4H8OS	104.2	909.9	264.103	1.0867	mono
22	Cyclohexen−2‐one	C930687	C6H8O	96.1	918	269.439	1.1098	
23	*N*‐nitrosodiethylamine	C55185	C4H10N2O	102.1	897.9	256.562	1.1479	
24	Benzaldehyde	C100527	C7H6O	106.1	963.7	304.911	1.1488	mono
25	Octanal	C124130	C8H16O	128.2	1,007	349.69	1.4063	mono
26	13C	unidentified	*	0	944.2	288.542	1.3026	
27	Benzaldehyde	C100527	C7H6O	106.1	962.8	304.102	1.4666	dimer
28	Heptanal	C111717	C7H14O	114.2	900.9	258.372	1.3292	mono
29	3‐methylthiopropanal	C3268493	C4H8OS	104.2	907.3	262.447	1.3961	dimer
30	14C	unidentified	*	0	858.9	234.396	1.1369	
31	15C	unidentified	*	0	877.5	244.54	1.2697	
32	16C	unidentified	*	0	900.6	258.209	1.4269	
33	17C	unidentified	*	0	788.2	200.514	1.3574	
34	18C	unidentified	*	0	789.1	200.913	1.398	
35	Furfural	C98011	C5H4O2	96.1	827.7	218.686	1.3297	
36	Pentan−1‐ol	C71410	C5H12O	88.1	763.3	189.603	1.2521	
37	3‐pentanone	C96220	C5H10O	86.1	694.4	163.468	1.1109	mono
38	*n*‐propyl acetate	C109604	C5H10O2	102.1	711.7	169.31	1.1648	mono
39	19C	unidentified	*	0	672.1	156.833	1.1616	
40	1‐butanol	C71363	C4H10O	74.1	647.6	150.411	1.1741	
41	20C	unidentified	*	0	729.4	175.826	1.186	
42	Pentanal	C110623	C5H10O	86.1	697.4	164.416	1.1858	
43	2‐butanone	C78933	C4H8O	72.1	587.4	136.814	1.2451	dimer
44	21C	unidentified	*	0	603.1	140.225	1.2916	
45	22C	unidentified	*	0	647.5	150.404	1.3275	
46	Acetic acid ethyl ester	C141786	C4H8O2	88.1	618.6	143.642	1.3379	dimer
47	2‐methyl−1‐propanol	C78831	C4H10O	74.1	636.8	147.822	1.3716	
48	3‐pentanone	C96220	C5H10O	86.1	693.5	163.194	1.3562	dimer
49	23C	unidentified	*	0	657.4	152.882	1.4039	
50	24C	unidentified	*	0	686.2	160.899	1.2675	
51	25C	unidentified	*	0	712.8	169.703	1.2969	
52	26C	unidentified	*	0	686.2	160.902	1.3127	
53	Hexanal	C66251	C6H12O	100.2	790.5	201.506	1.2571	mono
54	Acetic acid butyl ester	C123864	C6H12O2	116.2	806.2	208.622	1.2375	mono
55	27C	unidentified	*	0	750.5	184.257	1.3049	
56	28C	unidentified	*	0	712.8	169.703	1.4022	
57	29C	unidentified	*	0	701.6	165.824	1.4157	
58	30C	unidentified	*	0	702.6	166.16	1.4477	
59	*N*,*N*‐diethylethanamine	C121448	C6H15N	101.2	689.6	161.95	1.2289	
60	31C	unidentified	*	0	951.4	294.405	1.2635	
61	32C	unidentified	*	0	831.7	220.638	1.2091	
62	33C	unidentified	*	0	748.8	183.564	1.1066	
63	34C	unidentified	*	0	755.3	186.255	1.4113	
64	35C	unidentified	*	0	734.5	177.786	1.4245	
65	36C	unidentified	*	0	676.5	158.048	1.078	
66	37C	unidentified	*	0	788.2	200.488	1.469	
67	Hexanal	C66251	C6H12O	100.2	792.5	202.431	1.561	dimer
68	38C	unidentified	*	0	788.1	200.441	1.5285	
69	Acetic acid butyl ester	C123864	C6H12O2	116.2	805.9	208.485	1.6192	dimer
70	*n*‐Propyl acetate	C109604	C5H10O2	102.1	711.3	169.144	1.4752	dimer
71	39C	unidentified	*	0	690	162.078	1.573	
72	40C	unidentified	*	0	713.4	169.881	1.5378	
73	Ethyl 2‐hydroxypropanoate	C97643	C5H10O3	118.1	805.1	208.089	1.5362	
74	Octamethylcyclotetrasiloxane	C556672	C8H24O4Si4	296.6	1,009	352.022	1.6762	
75	Octanal	C124130	C8H16O	128.2	1,007.2	349.831	1.8216	dimer
76	41C	unidentified	*	0	1,006.7	349.269	1.4991	
77	42C	unidentified	*	0	980.6	320.885	1.1066	
78	Heptanal	C111717	C7H14O	114.2	901	258.418	1.6948	dimer
79	43C	unidentified	*	0	811.2	210.943	1.8259	
80	44C	unidentified	*	0	777.6	195.833	1.7521	
81	Isopropyl alcohol	C67630	C3H8O	60.1	523	122.845	1.219	dimer
82	45C	unidentified	*	0	737	178.771	1.285	
83	3‐hydroxybutan−2‐one	C513860	C4H8O2	88.1	712.9	169.706	1.328	dimer
84	46C	unidentified	*	0	904.1	260.356	1.528	
85	Nonanal	C124196	C9H18O	142.2	1,109.5	494.176	1.4776	
86	Compound from HS‐Vial Septum	GAS_00002	*	0	836	222.729	1.4664	
87	47C	unidentified	*	0	860.4	235.177	1.2009	
88	(E, E)−2,4‐heptadienal	C4313035	C7H10O	110.2	1,017.6	362.652	1.1919	
89	2‐heptanone	C110430	C7H14O	114.2	891	252.342	1.2602	
90	Furaneol	C3658773	C6H8O3	128.1	1,074.1	441.089	1.1985	
91	Linalool	C78706	C10H18O	154.3	1,093.8	470.625	1.217	
92	48C	unidentified	*	0	993.9	334.717	1.1761	
93	49C	unidentified	*	0	577.3	134.63	1.2639	
94	50C	unidentified	*	0	850	229.758	1.5154	
95	51C	unidentified	*	0	469.3	111.221	1.0941	
96	52C	unidentified	*	0	558.3	130.513	1.0884	
97	53C	unidentified	*	0	1,286.3	759.457	1.3455	
98	54C	unidentified	*	0	1,397.6	926.495	1.4833	
99	Nonanoic acid	C112050	C9H18O2	158.2	1,266.9	730.255	1.5409	
100	55C	unidentified	*	0	1,205.2	637.789	1.8124	

Note

CAS is the registration number of chemical substances by Chemical Abstracts Service.

^a^ Represents the retention time in the capillary GC column.

^b^ Represents the retention index calculated on FS‐SE‐54‐CB column using *n*‐ketones C4‐C9 as external standard.

^c^ Represents the drift time in the drift tube

### 3.5 Fingerprints of squid samples

**Figure 4:**
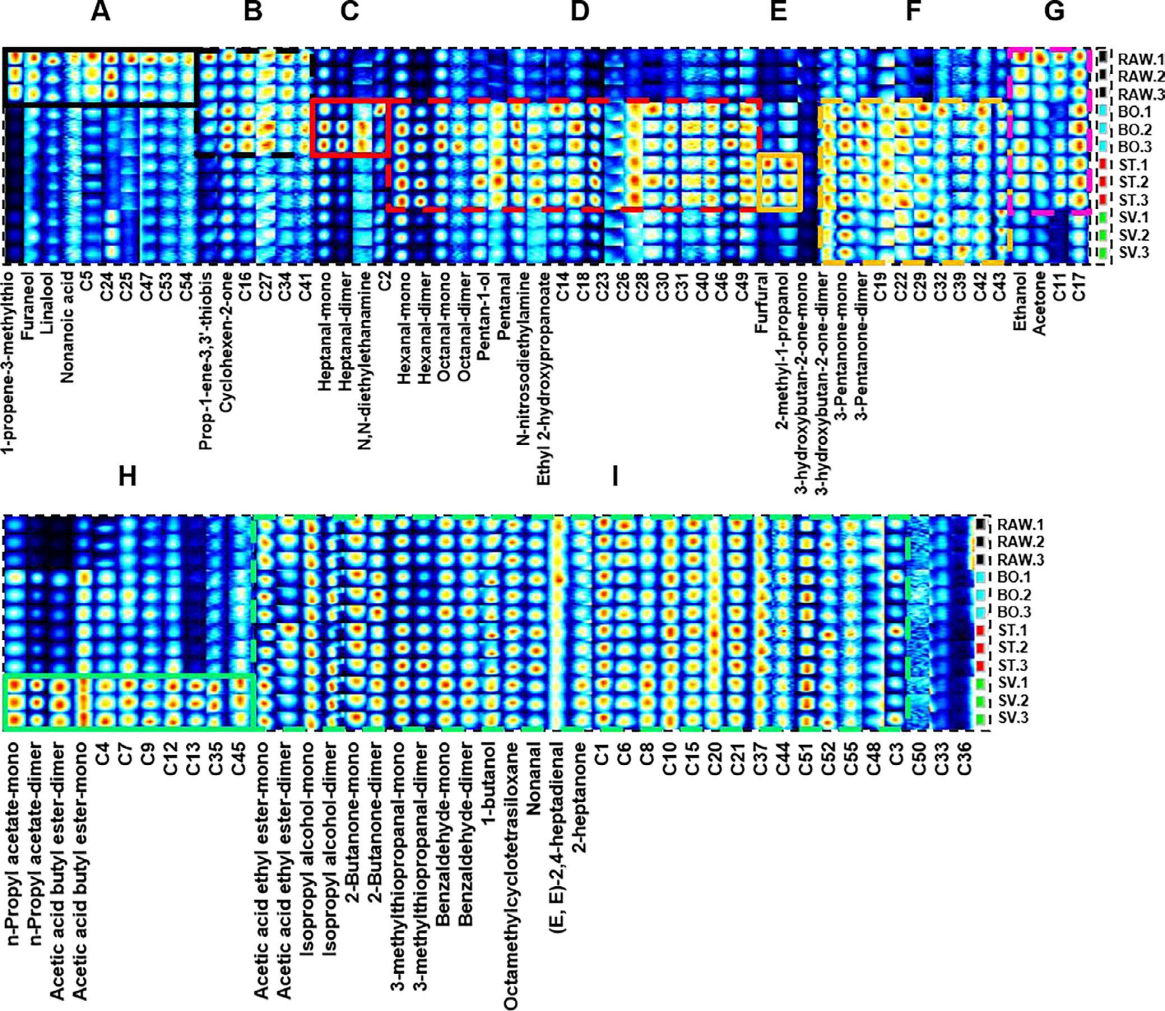
Fingerprint of squid in different cooking methods. RAW: raw squid; BO: boiled squid; ST: steamed squid; SV: sous vide cooked squid

In order to compare the differences in VOCs more comprehensively, the gallery plot plug‐in of LAV software was used to generate the peak signal of the topographic plots of squid samples (Figure 4). In Figure 4, each row represents all selected signal peaks from a squid sample, and each column represents the signal peak of the same volatile organic compound. Brightness indicates the content of a substance. The higher the brightness, the higher the content of the substance.

The complete VOC information for each sample and the differences between the samples are depicted in Figure 4. The material marked by the solid black line in area A has the highest content in RAW samples, such as 1‐propene‐3‐methylthio, furaneol, linalool, and nonanoic acid. The substances marked by the solid red line in area C had the highest content in the BO squid, such as heptanal and N, N‐diethylethanamine. The substances marked by the solid yellow line in area E were found in ST squid. The compounds with the highest content include furfural and 2‐methyl‐1‐propanol. The solid green line in region H had the highest content in SV squid, including compounds such as n‐propyl acetate and acetic acid ethyl ester.

The substances marked by the black dotted line in area B were the highest in RAW and BO squid, such as prop‐1‐ene‐3, 3'‐thiobis, and cyclohexen‐2‐one. The substances marked by the red dotted line in the area D were the highest in BO and ST squid, including hexanal, octanal, pentan‐1‐ol, pentanal, N‐nitrosodiethylamine, and ethyl 2‐hydroxypropanoate. The substances marked by the yellow dotted line in area F were the highest in BO, ST, and SV squid, such as 3‐hydroxybutan‐2‐one and 3‐pentanone. The substances marked by the red dotted line in the area G were the highest in RAW, BO, and ST squid, including ethanol and acetone. The green dashed line in area I indicates the flavor substances that were common to the four kinds of the samples, such as acetic acid ethyl ester, isopropyl alcohol, 2‐butanone, 3‐methylthiopropanal, benzaldehyde, 1‐butanol, octamethylcyclotetrasiloxane, nonanal, (E,E)‐2,4‐heptadienal, and 2‐heptanone.

**Figure 5:**
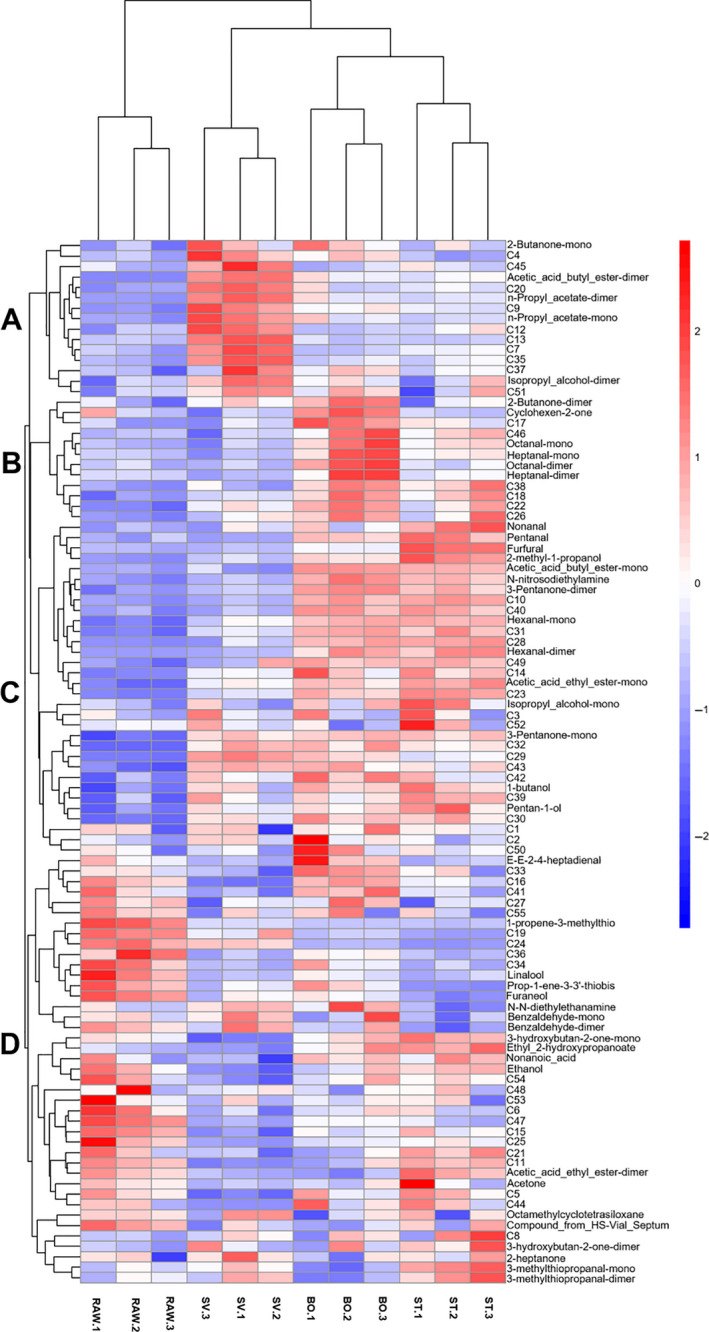
Heat map of squid from different cooking methods. RAW: raw squid; BO: boiled squid; ST: steamed squid; SV: sous vide cooked squid

The heat map is an intuitive and visual method for analyzing the distribution of experimental data. It can cluster data and samples to determine the quality of the samples (Metsalu & Vilo, 2015). There were four treatment groups: RAW, BO, ST, and SV. The VOCs were classified into four clusters: group A, group B, group C, and group D in Figure 5. The volatile compounds in group A were mainly represented in RAW. Volatile compounds in group B were mainly represented in BO and ST. Volatile compounds in group C were represented in BO, and volatile compounds in group D were only represented in SV. From Figure 5, SV samples had particular volatile components that are different from ST samples, BO samples, and RAW samples, such as acetic acid butyl ester, n‐propyl acetate‐mono, and n‐propyl acetate‐dimer. Also, BO samples and ST samples had acetic acid ethyl ester‐mono, hexanal‐mono, hexanal‐dimer, N‐nitrosodiethylamine, 3‐pentanone‐dimer, and acetic acid butyl ester‐mono. This result was different from SV samples. Some unknown volatile compounds need to be further studied and determined.

## 4 DISCUSSION

Sensory evaluation is one of the critical indicators of food quality. In this study, the sensory evaluation could distinguish the squid samples with different cooking methods. The squid samples did not need other pretreatments, so only the professionalism and consistency of the evaluation were considered in the sensory assessment, ignoring the importance of consumers’ demand for the product. Consumers’ perceptions can be used for the assessment in the product development, which can significantly save time and labor costs (Vieira et al., 2020). It also suggested that evaluation based on preferred attribute elicitation methodology had the advantage of providing data on the importance of attributes for product acceptance (da Costa et al., 2020). Besides, in the sensory evaluation, the combination of the projective method (Judacewski et al., 2019), descriptive analysis method (H. Silva et al., 2018), and temporal methods (de Souza Paglarini et al., 2020) is more conducive to the development of new squid products.

The aroma is one of the most important evaluation indicators of meat products. Aroma contributes to the acceptability of the meat, and it is the result of a combination of various volatile compounds and chemical reactions that form flavor components, including Maillard reactions, lipid oxidation, interactions between Maillard reaction products, and lipid oxidation products (Shahidi, 1998). Different cooking methods affect the flavor of meat products (Grosch, 1982), especially the volatile components (Macleod, Seyyedain‐Ardebili, & Chang, 1981). The types of VOCs present in the BO and ST samples were similar. These techniques utilize the same temperature and pressure. SV heats the sample for a long time, and it utilizes low temperature under vacuum conditions. Thus, the VOCs are significantly different from BO and ST. Studies have shown that different heating methods affect the quality of food (F. A. Silva, Ferreira, Madruga, & Estevez, 2016). Even for SV, product quality is different under different vacuum degrees (Jeong, O, Shin, & Kim, 2018).

Volatile compounds of cooked meat include products of lipid and fatty acid oxidation, for example, aliphatic hydrocarbons, aldehydes, ketones, alcohols, carboxylic acids, and esters (Mottram, 1998). Aldehydes are the main products of the oxidative degradation of fatty acids and the characteristic aroma components of meat (Mottram, 1998). In this study, 43 VOCs were identified, including 14 aldehydes. The threshold value of aldehydes is relatively low, which contributes significantly to the flavor in cooked squid. In the experiment, 3‐methylthiopropanal was present in all four sample types. 3‐Methylthioprapanol was first identified as a volatile component in cooked squid by Kubota, Matsukage, Sekiwa, and Kobayashi (1996) It is a “baked potato” descriptor and considered an essential contributor to the scent of squid (Carrascon, Escudero, Ferreira, & Lopez, 2014) as well as cooked lobster (Lee, Suriyaphan, & Cadwallader, 2001). However, the difference in the contents of aldehydes in squid from different heating methods has an important impact on the sample odor. Ketones are also products of fatty acid oxidation, generally the products of automatic oxidation of unsaturated fatty acids (Thomas, 1971). There are ten ketones in the 43 VOCs detected in ST, BO, and SV samples, such as 3‐hydroxybutan‐2‐one, 3‐pentanone, acetone, 2‐butanone, and 2‐heptanone. In particular, furaneol (4‐hydroxy‐2,5‐dimethyl‐3(2H)furanone) in RAW samples is described as a “caramel‐like” flavor, and it is a flavor component of strawberry (Buechi, Demole, & Thomas, 1973) and pineapple (Rodin, Himel, Silverstein, Leeper, & Gortner, 1965). Kubota et al. (1996) considered furaneol a substance that contributes to the sweetness of squid after cooking. Ester compounds are produced by the esterification of alcohols and acids (Domínguez, Gómez, Fonseca, & Lorenzo, 2014) and esters are found in many flavors of crustacean fish products that are cooked and heated (Tanchotikul & Hsieh, 1989). SV squid has the highest content of n‐propyl acetate and acetic acid ethyl ester of all samples tested. The volatile substance n‐propyl acetate is a short‐chain aliphatic ester that produces a pleasant fruity aroma. It is a component of natural flavors (Mahapatra, Kumari, Garlapati, Banerjee, & Nag, 2009). This indicates the SV con ditions are conducive to the esterification reaction and can yield more desirable flavor in squid.

The type of volatile components was determined by the degree of oxidative degradation of fatty acids under different heating temperatures, heating pressures, and transfer medium. The results show that the aldehydes were more varied and in higher abundance when squid was heated at 100°C under normal pressure. Conversely, the aldehydes of SV were less abundant than ST and BO. The reason is high‐temperature and high‐pressure conditions are conducive to the thermal oxidative degradation of fatty acids (Cheah & Ledward, 1996), and more volatile components are generated when heated. The increase in heating temperature increased the degree of oxidative degradation of fatty acids, thus increasing the content of volatile components. Compared with ST and BO at normal pressure and 100°C, SV is heated under vacuum at 60°C, and the degree of fatty acid oxidation degradation is lower during heating (Oz & Zikirov, 2015). This reduces the content of volatile substances generated during heating. Falowo, Muchenje, and Hugo (2017) reported that there was no pronounced effect of SV cooking temperature on fatty acid of beef and liver compared to raw samples. Generally, low heating temperature is indeed a weakness of SV cooking, but long‐term heating sometimes compensates for this weakness. Long‐time heating increases the volatile substances derived from the degradation of amino acids and/or thiamine (Roldan, Antequera, Armenteros, & Ruiz, 2014; Roldan, Ruiz, del Pulgar, Perez‐Palacios, & Antequera, 2015), such as 2‐methyl‐thiophene, carbon disulfide, benzothiazole, and dimethyl disulfide (Calkins & Hodgen, 2007). Mortensen, Frøst, Skibsted, and Risbo (2012) indicated that the effect of SV heating time on flavor has a greater effect than the heating temperature. For beef and pork cooked in SV, the longer the cooking time, the better the flavor (Christensen et al., 2012). SV cooking still develops a pleasant flavor at a low temperature. 3‐Methylbutanal, a meaty‐nutty flavor of dry‐cured ham (Ruiz, Ventanas, Cava, Andrés, & García, 1999), was found in SV cooked meat after 24 hr of heating at 60°C (del Pulgar, Roldan, & Ruiz‐Carrascal, 2013; Roldan, Ruiz, et al., 2015). Of course, the prolonged heating time of SV reduces the lipid oxidation of carbonyl compounds in food, which indicates that they further react with other compounds (proteins, amino acids, etc.) to produce new, more desirable volatiles (del Pulgar et al., 2013; Roldan, Ruiz, et al., 2015). In this study, n‐propyl acetate and acetic acid ethyl ester (highlighted in group H in Figure 4) were produced after SV heating; however, they were not detected in BO and ST. Moreover, by adding reducing sugar or other flavor precursors, SV food flavor can also be enhanced (Roldan, Loebner, et al., 2015).

In addition to this, SV technology produces high‐quality sensory characteristics of meat products. Studies have shown that a slow heating rate is key to generating tender meat (Cover, 1943) and maintaining the meat core temperature closed to 60°C for a long time (Laakkonen, Sherbon, & Wellington, 1970). These observations have been further confirmed in the SV technique (Becker et al., 2015; Christensen et al., 2013; Roldan et al., 2013). SV means not only higher sensorial quality but LTLT heating can also minimize the nutrient loss (Rondanelli et al., 2017), improve bioaccessibility (da Silva et al., 2017), ensure the safety of food (El Kadri, Alaizoki, Celen, Smith, & Onyeaka, 2020; Nissen, Rosnes, Brendehaug, & Kleiberg, 2002), and extend the shelf life (Diaz, Nieto, Banon, & Garrido, 2009; Kim, Hong, Lim, Park, & Lee, 2015; Nissen et al., 2002). Given the many advantages of SV and the growing demand for nutritious and convenient foods, the application of SV technology in the industrial production of squid is feasible.

## 5 CONCLUSIONS

Through sensory evaluation and electronic analysis, it was found that different cooking methods have a great influence on the flavor of the squid. SV squid flavor is different from that of ST and BO. Additionally, a method was developed to evaluate the characteristic volatile compounds of squid samples from different cooking methods by establishing the fingerprint with HS‐GC‐IMS. A total of 43 volatile substances, including some dimers, were detected by GC‐IMS analysis from samples of squid from different cooking methods. The predominant compounds were mainly aldehydes, ketones, alcohols, and esters. Given the many advantages of SV technology, it could be used for the industrial production of squid.

## 6 INFORMED CONSENT

Written informed consent was obtained from all study participants.

## CONFLICTS OF INTEREST

The authors declare that they do not have any conflict of interest.

## AUTHORS CONTRIBUTION

Hao Zhang and Zhenkun Cui conceived the study; Zhenkun Cui, Hongbo Li, and Tatiana Manoli designed the methodology; Han Yan provided the software; Zhenkun Cui, Han Yan, and Hongbo Li involved in formal analysis; Zhenkun Cui and Han Yan wrote the original draft of the manuscript; Haizhen Mo and Tatiana Manoli wrote, reviewed, and edited the manuscript; Haizhen Mo and Hao Zhang involved in funding acquisition; and Hao Zhang investigated twehe study. All authors have read and agree to the published version of the manuscript.

## ETHICAL APPROVAL

This study does not involve any human or animal testing.

## References

[fsn31877-bib-0001] Alemán, A. , Giménez, B. , Pérez‐Santin, E. , Gómez‐Guillén, M. , & Montero, P. (2011). Contribution of Leu and Hyp residues to antioxidant and ACE‐inhibitory activities of peptide sequences isolated from squid gelatin hydrolysate. Food Chemistry, 125(2), 334–341. 10.1016/j.foodchem.2010.08.058

[fsn31877-bib-0002] Alemán, A. , Gómez‐Guillén, M. , & Montero, P. (2013). Identification of ace‐inhibitory peptides from squid skin collagen after in vitro gastrointestinal digestion. Food Research International, 54(1), 790–795. 10.1016/j.foodres.2013.08.027

[fsn31877-bib-0003] Armenta, S. , Alcala, M. , & Blanco, M. (2011). A review of recent, unconventional applications of ion mobility spectrometry (IMS). Analytica Chimica Acta, 703(2), 114–123. 10.1016/j.aca.2011.07.021 21889625

[fsn31877-bib-0004] Asbury, G. R. , Klasmeier, J. , & Hill, H. H. Jr (2000). Analysis of explosives using electrospray ionization/ion mobility spectrometry (ESI/IMS). Talanta, 50(6), 1291–1298. 10.1016/S0039-9140(99)00241-6 18967826

[fsn31877-bib-0005] Baldwin, D. E. (2012). Sous vide cooking: A review. International Journal of Gastronomy Food Science, 1(1), 15–30. 10.1016/j.ijgfs.2011.11.002

[fsn31877-bib-0006] Becker, A. , Boulaaba, A. , Pingen, S. , Röhner, A. , & Klein, G. (2015). Low temperature, long time treatment of porcine M. longissimus thoracis et lumborum in a combi steamer under commercial conditions. Meat Science, 110, 230–235. 10.1016/j.meatsci.2015.07.024 26263040

[fsn31877-bib-0007] Botinestean, C. , Keenan, D. F. , Kerry, J. P. , & Hamill, R. M. (2016). The effect of thermal treatments including sous‐vide, blast freezing and their combinations on beef tenderness of M. semitendinosus steaks targeted at elderly consumers. LWT‐Food Science and Technology, 74, 154–159. 10.1016/j.lwt.2016.07.026

[fsn31877-bib-0008] Buechi, G. , Demole, E. , & Thomas, A. F. (1973). Syntheses of 2, 5‐dimethyl‐4‐hydroxy‐2, 3‐dihydrofuran‐3‐one (furaneol), a flavor principle of pineapple and strawberry. The Journal of Organic Chemistry, 38(1), 123–125. 10.1021/jo00941a025

[fsn31877-bib-0009] Calkins, C. R. , & Hodgen, J. M. (2007). A fresh look at meat flavor. Meat Science, 77(1), 63–80. 10.1016/j.meatsci.2007.04.016 22061397

[fsn31877-bib-0010] Carrascon, V. , Escudero, A. , Ferreira, V. , & Lopez, R. (2014). Characterisation of the key odorants in a squid broth (Illex argentinus). LWT‐Food Science and Technology, 57(2), 656–662. 10.1016/j.lwt.2014.02.010

[fsn31877-bib-0011] Cheah, P. , & Ledward, D. (1996). High pressure effects on lipid oxidation in minced pork. Meat Science, 43(2), 123–134. 10.1016/0309-1740(96)84584-0 22060567

[fsn31877-bib-0012] Christensen, L. , Ertbjerg, P. , Løje, H. , Risbo, J. , van den Berg, F. W. , & Christensen, M. (2013). Relationship between meat toughness and properties of connective tissue from cows and young bulls heat treated at low temperatures for prolonged times. Meat Science, 93(4), 787–795. 10.1016/j.meatsci.2012.12.001 23305828

[fsn31877-bib-0013] Christensen, L. , Gunvig, A. , Tørngren, M. A. , Aaslyng, M. D. , Knøchel, S. , & Christensen, M. (2012). Sensory characteristics of meat cooked for prolonged times at low temperature. Meat Science, 90(2), 485–489. 10.1016/j.meatsci.2011.09.012 21985894

[fsn31877-bib-0014] Cover, S. (1943). Effect of extremely low rates of heat penetration on tendering of beef. Food Research, 8, 388–394. 10.1111/j.1365-2621.1943.tb16573.x

[fsn31877-bib-0015] Cross, H. , Stanfield, M. S. , & Koch, E. J. (1976). Beef palatability as affected by cooking rate and final internal temperature. Journal of Animal Science, 43(1), 114–121. 10.2527/jas1976.431114x

[fsn31877-bib-0016] Cui, Z. , Dubova, H. , & Mo, H. (2019). Effects of sous vide cooking on physicochemical properties of squid. Journal of Hygienic Engineering and Design, 29, 35–40.

[fsn31877-bib-0017] da Costa, G. M. , de Paula, M. M. , Costa, G. N. , Esmerino, E. A. , Silva, R. , de Freitas, M. Q. , … Pimentel, T. C. (2020). Preferred attribute elicitation methodology compared to conventional descriptive analysis: A study using probiotic yogurt sweetened with xylitol and added with prebiotic components. Journal of Sensory Studies, e12602, 10.1111/joss.12602

[fsn31877-bib-0018] da Silva, F. L. F. , de Lima, J. P. S. , Melo, L. S. , da Silva, Y. S. M. , Gouveia, S. T. , Lopes, G. S. , & Matos, W. O. (2017). Comparison between boiling and vacuum cooking (sous‐vide) in the bioaccessibility of minerals in bovine liver samples. Food Research International, 100, 566–571. 10.1016/j.foodres.2017.07.054 28873722

[fsn31877-bib-0019] de Souza Paglarini, C. , Vidal, V. A. S. , dos Santos, M. , Coimbra, L. O. , Esmerino, E. A. , Cruz, A. G. , & Pollonio, M. A. R. (2020). Using dynamic sensory techniques to determine drivers of liking in sodium and fat‐reduced Bologna sausage containing functional emulsion gels. Food Research International, 132, 109066 10.1016/j.foodres.2020.109066 32331676

[fsn31877-bib-0020] del Pulgar, J. S. , Gazquez, A. , & Ruiz‐Carrascal, J. (2012). Physico‐chemical, textural and structural characteristics of sous‐vide cooked pork cheeks as affected by vacuum, cooking temperature, and cooking time. Meat Science, 90(3), 828–835. 10.1016/j.meatsci.2011.11.024 22154568

[fsn31877-bib-0021] del Pulgar, J. S. , Roldan, M. , & Ruiz‐Carrascal, J. (2013). Volatile Compounds Profile of Sous‐Vide Cooked Pork Cheeks as Affected by Cooking Conditions (Vacuum Packaging, Temperature and Time). Molecules, 18(10), 12538–12547. 10.3390/molecules181012538 24152673 PMC6270416

[fsn31877-bib-0022] Diaz, P. , Nieto, G. , Banon, S. , & Garrido, M. D. (2009). Determination of Shelf Life of Sous Vide Salmon (Salmo Salard) Based on Sensory Attributes. Journal of Food Science, 74(8), S371–S376. 10.1111/j.1750-3841.2009.01317.x 19799682

[fsn31877-bib-0023] Domínguez, R. , Gómez, M. , Fonseca, S. , & Lorenzo, J. M. (2014). Influence of thermal treatment on formation of volatile compounds, cooking loss and lipid oxidation in foal meat. LWT‐Food Science and Technology, 58(2), 439–445. 10.1016/j.lwt.2014.04.006 24583332

[fsn31877-bib-0024] Dong, L. , Zhu, J. , Li, X. , & Li, J. (2013). Effect of tea polyphenols on the physical and chemical characteristics of dried‐seasoned squid (*Dosidicus gigas*) during storage. Food Control, 31(2), 586–592. 10.1016/j.foodcont.2012.10.014

[fsn31877-bib-0025] Eiceman, G. A. , Karpas, Z. , & Hill, H. H. Jr (2013). Ion mobility spectrometry. Boca Raton, FL: CRC Press.

[fsn31877-bib-0026] El Kadri, H. , Alaizoki, A. , Celen, T. , Smith, M. , & Onyeaka, H. (2020). The effect of low‐temperature long‐time (LTLT) cooking on survival of potentially pathogenic Clostridium perfringens in beef. International Journal of Food Microbiology, 320, 108540 10.1016/j.ijfoodmicro.2020.108540 32044624

[fsn31877-bib-0027] Falowo, A. B. , Muchenje, V. , & Hugo, A. (2017). Effect of sous‐vide technique on fatty acid and mineral compositions of beef and liver from Bonsmara and non‐descript cattle. Annals of Animal Science, 17(2), 565–580. 10.1515/aoas-2016-0078

[fsn31877-bib-0028] Garrido‐Delgado, R. , Muñoz‐Pérez, M. E. , & Arce, L. (2018). Detection of adulteration in extra virgin olive oils by using UV‐IMS and chemometric analysis. Food Control, 85, 292–299. 10.1016/j.foodcont.2017.10.012

[fsn31877-bib-0029] Gloess, A. , Yeretzian, C. , Knochenmuss, R. , & Groessl, M. (2018). On‐line analysis of coffee roasting with ion mobility spectrometry–mass spectrometry (IMS–MS). International Journal of Mass Spectrometry, 424, 49–57. 10.1016/j.ijms.2017.11.017

[fsn31877-bib-0030] Gou, J. , Lee, H.‐Y. , & Ahn, J. (2010). Effect of high pressure processing on the quality of squid (*Todarodes pacificus*) during refrigerated storage. Food Chemistry, 119(2), 471–476. 10.1016/j.foodchem.2009.06.042

[fsn31877-bib-0031] Grosch, W. (1982). Lipid degradation products and flavour In Morton I. D., & MacLeod A. J. (Eds.), Food Flavours (pp. 325–398). Amsterdam: Elsevier Scientific Publication.

[fsn31877-bib-0032] Hansen, T. B. , Knøchel, S. , Juncher, D. , & Bertelsen, G. (1995). Storage characteristics of sous vide cooked roast beef. International Journal of Food Science & Technology, 30(3), 365–378. 10.1111/j.1365-2621.1995.tb01384.x

[fsn31877-bib-0033] Ivanov, G. , Bilgucu, E. , Ivanova, I. , & Dimitrova, M. (2020). Volatile organic compound profiles of yoghurt produced from cow's milk with different somatic cell counts. International Journal of Dairy Technology, 10.1111/1471-0307.12702

[fsn31877-bib-0034] Jeong, K. , O, H. , Shin, S. Y. , & Kim, Y.‐S. (2018). Effects of sous‐vide method at different temperatures, times and vacuum degrees on the quality, structural, and microbiological properties of pork ham. Meat Science, 143, 1–7. 10.1016/j.meatsci.2018.04.010 29684839

[fsn31877-bib-0035] Jo, D. , Kim, G.‐R. , Yeo, S.‐H. , Jeong, Y.‐J. , Noh, B. S. , & Kwon, J.‐H. (2013). Analysis of aroma compounds of commercial cider vinegars with different acidities using SPME/GC‐MS, electronic nose, and sensory evaluation. Food Science and Biotechnology, 22(6), 1559–1565. 10.1007/s10068-013-0251-1

[fsn31877-bib-0036] Judacewski, P. , Los, P. R. , Lima, L. S. , Alberti, A. , Zielinski, A. A. F. , & Nogueira, A. (2019). Perceptions of Brazilian consumers regarding white mould surface‐ripened cheese using free word association. International Journal of Dairy Technology, 72(4), 585–590. 10.1111/1471-0307.12649

[fsn31877-bib-0037] Karpas, Z. , Guamán, A. V. , Calvo, D. , Pardo, A. , & Marco, S. (2012). The potential of ion mobility spectrometry (IMS) for detection of 2, 4, 6‐trichloroanisole (2, 4, 6‐TCA) in wine. Talanta, 93, 200–205. 10.1016/j.talanta.2012.02.012 22483899

[fsn31877-bib-0038] Kim, J. H. , Hong, G. E. , Lim, K. W. , Park, W. , & Lee, C. H. (2015). Influence of citric acid on the pink color and characteristics of sous vide processed chicken breasts during chill storage. Korean Journal for Food Science of Animal Resources, 35(5), 585–596. 10.5851/kosfa.2015.35.5.585 26761885 PMC4670886

[fsn31877-bib-0039] Kubota, K. , Matsukage, Y. , Sekiwa, Y. , & Kobayashi, A. (1996). Identification of the characteristic volatile flavor compounds formed by cooking squid (*Todarodes pacificus* Steenstrup). Food Science and Technology International, Tokyo, 2(3), 163–166. 10.3136/fsti9596t9798.2.163

[fsn31877-bib-0040] Laakkonen, E. , Sherbon, J. W. , & Wellington, G. H. (1970). Low‐temperature, long‐time heating of bovine muscle 3. Collagenolytic activity. Journal of Food Science, 35(2), 181–184. 10.1111/j.1365-2621.1970.tb12133.x

[fsn31877-bib-0041] Lee, G.‐H. , Suriyaphan, O. , & Cadwallader, K. R. (2001). Aroma components of cooked tail meat of American lobster (Homarus americanus). Journal of Agricultural and Food Chemistry, 49(9), 4324–4332. 10.1021/jf001523t 11559132

[fsn31877-bib-0042] Lyberg, A. M. , & Adlercreutz, P. (2008). Lipase‐catalysed enrichment of DHA and EPA in acylglycerols resulting from squid oil ethanolysis. European Journal of Lipid Science and Technology, 110(4), 317–324. 10.1002/ejlt.200700217

[fsn31877-bib-0043] Macleod, G. , Seyyedain‐Ardebili, M. , & Chang, S. S. (1981). Natural and simulated meat flavors (with particular reference to beef). Critical Reviews in Food Science & Nutrition, 14(4), 309–437. 10.1080/10408398109527309 7023846

[fsn31877-bib-0044] Mahapatra, P. , Kumari, A. , Garlapati, V. K. , Banerjee, R. , & Nag, A. (2009). Enzymatic synthesis of fruit flavor esters by immobilized lipase from Rhizopus oligosporus optimized with response surface methodology. Journal of Molecular Catalysis B: Enzymatic, 60(1–2), 57–63. 10.1016/j.molcatb.2009.03.010

[fsn31877-bib-0045] Metsalu, T. , & Vilo, J. (2015). ClustVis: A web tool for visualizing clustering of multivariate data using Principal Component Analysis and heatmap. Nucleic Acids Research, 43(W1), W566–W570. 10.1093/nar/gkv468 25969447 PMC4489295

[fsn31877-bib-0046] Mirzaei, H. , & Regnier, F. (2006). Identification and quantification of protein carbonylation using light and heavy isotope labeled Girard's P reagent. Journal of Chromatography A, 1134(1–2), 122–133. 10.1016/j.chroma.2006.08.096 16996067

[fsn31877-bib-0047] Mortensen, L. M. , Frøst, M. B. , Skibsted, L. H. , & Risbo, J. (2012). Effect of time and temperature on sensory properties in low‐temperature long‐time sous‐vide cooking of beef. Journal of Culinary Science & Technology, 10(1), 75–90. 10.1080/15428052.2012.651024

[fsn31877-bib-0048] Mottram, D. S. (1998). Flavour formation in meat and meat products: A review. Food Chemistry, 62(4), 415–424. 10.1016/S0308-8146(98)00076-4

[fsn31877-bib-0049] Nissen, H. , Rosnes, J. T. , Brendehaug, J. , & Kleiberg, G. H. (2002). Safety evaluation of sous vide‐processed ready meals. Letters in Applied Microbiology, 35(5), 433–438. 10.1046/j.1472-765x.2002.01218.x 12390496

[fsn31877-bib-0050] Oz, F. , & Zikirov, E. (2015). The effects of sous‐vide cooking method on the formation of heterocyclic aromatic amines in beef chops. LWT‐Food Science and Technology, 64(1), 120–125. 10.1016/j.lwt.2015.05.050

[fsn31877-bib-0051] Rearden, P. , & Harrington, P. B. (2005). Rapid screening of precursor and degradation products of chemical warfare agents in soil by solid‐phase microextraction ion mobility spectrometry (SPME–IMS). Analytica Chimica Acta, 545(1), 13–20. 10.1016/j.aca.2005.04.035

[fsn31877-bib-0052] Rodin, J. , Himel, C. M. , Silverstein, R. M. , Leeper, R. W. , & Gortner, W. A. (1965). Volatile Flavor and Aroma Components of Pineapple. l. Isolation and Tentative Identification of 2,5‐Dimethyl‐4‐Hydroxy‐3(2H)‐Furanone. Journal of Food Science, 30(2), 280–285. 10.1111/j.1365-2621.1965.tb00302.x

[fsn31877-bib-0053] Roldan, M. , Antequera, T. , Armenteros, M. , & Ruiz, J. (2014). Effect of different temperature‐time combinations on lipid and protein oxidation of sous‐vide cooked lamb loins. Food Chemistry, 149, 129–136. 10.1016/j.foodchem.2013.10.079 24295686

[fsn31877-bib-0054] Roldan, M. , Antequera, T. , Martin, A. , Mayoral, A. I. , & Ruiz, J. (2013). Effect of different temperature‐time combinations on physicochemical, microbiological, textural and structural features of sous‐vide cooked lamb loins. Meat Science, 93(3), 572–578. 10.1016/j.meatsci.2012.11.014 23273466

[fsn31877-bib-0055] Roldan, M. , Antequera, T. , Perez‐Palacios, T. , & Ruiz, J. (2014). Effect of added phosphate and type of cooking method on physico‐chemical and sensory features of cooked lamb loins. Meat Science, 97(1), 69–75. 10.1016/j.meatsci.2014.01.012 24530991

[fsn31877-bib-0056] Roldan, M. , Loebner, J. , Degen, J. , Henle, T. , Antequera, T. , & Ruiz‐Carrascal, J. (2015). Advanced glycation end products, physico‐chemical and sensory characteristics of cooked lamb loins affected by cooking method and addition of flavour precursors. Food Chemistry, 168, 487–495. 10.1016/j.foodchem.2014.07.100 25172739

[fsn31877-bib-0057] Roldan, M. , Ruiz, J. , del Pulgar, J. S. , Perez‐Palacios, T. , & Antequera, T. (2015). Volatile compound profile of sous‐vide cooked lamb loins at different temperature‐time combinations. Meat Science, 100, 52–57. 10.1016/j.meatsci.2014.09.010 25306511

[fsn31877-bib-0058] Rondanelli, M. , Daglia, M. , Meneghini, S. , Di Lorenzo, A. , Peroni, G. , Faliva, M. A. , & Perna, S. (2017). Nutritional advantages of sous‐vide cooking compared to boiling on cereals and legumes: Determination of ashes and metals content in ready‐to‐eat products. Food Science & Nutrition, 5(3), 827–833. 10.1002/fsn3.469 28572974 PMC5448368

[fsn31877-bib-0059] Ruiz, J. , Ventanas, J. , Cava, R. , Andrés, A. , & García, C., (1999). Volatile compounds of dry‐cured Iberian ham as affected by the length of the curing process. Meat Science, 52(1), 19–27. 10.1016/S0309-1740(98)00144-2 22062139

[fsn31877-bib-0060] Salaseviciene, A. , Vaiciulyte‐Funk, L. , & Koscelkovskienė, I. (2014). Impact of low temperature, prolonged time treatment and vacuum depth on the porcine muscle quality and safety. Paper presented at the 9th Baltic Conference on Food Science and Technology “Food for Consumer Well‐Being”

[fsn31877-bib-0061] Schellekens, M. (1996). New research issues in sous‐vide cooking. Trends in Food Science and Technology, 7(8), 256–262. 10.1016/0924-2244(96)10027-3

[fsn31877-bib-0062] Shahidi, F. (1998). Flavor of meat, meat products and seafoods. London: Blackie Academic and Professional.

[fsn31877-bib-0063] Shahidi, F. , Rubin, L. J. , D'Souza, L. A. , Teranishi, R. , & Buttery, R. G. (1986). Meat flavor volatiles: A review of the composition, techniques of analysis, and sensory evaluation. Critical Reviews in Food Science & Nutrition, 24(2), 141–243. 10.1080/10408398609527435 3527563

[fsn31877-bib-0064] Silva, F. A. , Ferreira, V. C. , Madruga, M. S. , & Estevez, M. (2016). Effect of the cooking method (grilling, roasting, frying and sous‐vide) on the oxidation of thiols, tryptophan, alkaline amino acids and protein cross‐linking in jerky chicken. Journal of Food Science and Technology, 53(8), 3137–3146. 10.1007/s13197-016-2287-8 27784908 PMC5055878

[fsn31877-bib-0065] Silva, H. , Balthazar, C. , Silva, R. , Vieira, A. , Costa, R. , Esmerino, E. , … Cruz, A. (2018). Sodium reduction and flavor enhancer addition in probiotic prato cheese: Contributions of quantitative descriptive analysis and temporal dominance of sensations for sensory profiling. Journal of Dairy Science, 101(10), 8837–8846. 10.3168/jds.2018-14819 30077456

[fsn31877-bib-0066] Snyder, A. P. , Harden, C. S. , Davis, D. M. , Shoff, D. B. , & Maswadeh, W. M. (1995). Hand‐portable Gas Chromatography‐Ion Mobility spectrometer for the determination of the freshness of fish. Paper presented at the Third International Workshop on Ion Mobility Spectrometry, Galveston, Texas, USA.

[fsn31877-bib-0067] Tanchotikul, U. , & Hsieh, T. C. Y. (1989). Volatile flavor components in crayfish waste. Journal of Food Science, 54(6), 1515–1520. 10.1111/j.1365-2621.1989.tb05149.x

[fsn31877-bib-0068] Thomas, C. (1971). Sources of flavor in poultry skin. Food Technol, 25, 109–115.

[fsn31877-bib-0069] Tomac, A. , Cova, M. C. , Narvaiz, P. , & Yeannes, M. I. (2017). Sensory acceptability of squid rings gamma irradiated for shelf‐life extension. Radiation Physics and Chemistry, 130, 359–361. 10.1016/j.radphyschem.2016.09.016

[fsn31877-bib-0070] Vaudagna, S. R. , Sánchez, G. , Neira, M. S. , Insani, E. M. , Picallo, A. B. , Gallinger, M. M. , & Lasta, J. A. (2002). Sous vide cooked beef muscles: Effects of low temperature–long time (LT–LT) treatments on their quality characteristics and storage stability. International Journal of Food Science and Technology, 37(4), 425–441. 10.1046/j.1365-2621.2002.00581.x

[fsn31877-bib-0071] Verkouteren, J. R. , & Staymates, J. L. (2011). Reliability of ion mobility spectrometry for qualitative analysis of complex, multicomponent illicit drug samples. Forensic Science International, 206(1–3), 190–196. 10.1016/j.forsciint.2010.08.005 20828951

[fsn31877-bib-0072] Vieira, A. H. , Balthazar, C. F. , Rocha, R. S. , Silva, R. , Guimaraes, J. T. , Pagani, M. M. , … Tonon, R. V. (2020). The free listing task for describing the sensory profiling of dairy foods: A case study with microfiltered goat whey orange juice beverage. Journal of Sensory Studies, e12594, 10.1111/joss.12594

[fsn31877-bib-0073] Waluda, C. , Rodhouse, P. , Podestá, G. , Trathan, P. , & Pierce, G. (2001). Surface oceanography of the inferred hatching grounds of Illex argentinus (Cephalopoda: Ommastrephidae) and influences on recruitment variability. Marine Biology, 139(4), 671–679. 10.1007/s002270100615

[fsn31877-bib-0074] Wu, Z. , Chen, L. , Wu, L. , Xue, X. , Zhao, J. , Li, Y. , … Lin, G. (2015). Classification of Chinese honeys according to their floral origins using elemental and stable isotopic compositions. Journal of Agricultural and Food Chemistry, 63(22), 5388–5394. 10.1021/acs.jafc.5b01576 25990572

